# PREDNISOLONE EYE DROPS AS A POTENTIAL TREATMENT IN NONNEOVASCULAR PACHYCHOROID-RELATED DISEASES

**DOI:** 10.1097/IAE.0000000000004109

**Published:** 2024-03-18

**Authors:** Femke M. van den Tillaart, Irene M. Temmerman, Franca Hartgers, Suzanne Yzer

**Affiliations:** Department of Ophthalmology, Radboud University Medical Center, Nijmegen, The Netherlands.

**Keywords:** central serous chorioretinopathy, pachychoroid, pachychoroid disease spectrum, pachychoroid pigment epitheliopathy, peripapillary pachychoroid syndrome, prednisolone eye drops

## Abstract

**Purpose:**

To investigate the functional and structural outcomes after treatment with prednisolone eye drops in the following pachychoroid-related diseases: chronic central serous chorioretinopathy, pachychoroid pigment epitheliopathy, and peripapillary pachychoroid syndrome.

**Methods:**

In this retrospective study, 54 eyes of 48 patients with pachychoroid-related disease were treated with prednisolone acetate 1% eye drops 3 times a day. Change in macular volume and retinal central subfield thickness on optical coherence tomography was measured. In addition, the foveal or complete resolution of fluid and the change in visual acuity were studied.

**Results:**

The follow-up visit was at a mean of 41.2 ± 14.5 days. In the 44 eyes with chronic central serous chorioretinopathy, a significant reduction in retinal central subfield thickness (*P* < 0.001) and macular volume (*P* < 0.001) was observed. Foveal intra- or subretinal fluid resolved completely in 22% of the eyes. In the 8 peripapillary pachychoroid syndrome eyes, a reduction in the nasal retinal thickness was observed (*P* = 0.025). One of the 2 pachychoroid pigment epitheliopathy eyes showed structural improvement. No significant change in visual acuity was observed in any of the pachychoroid spectrum diseases.

**Conclusion:**

In patients with chronic central serous chorioretinopathy, peripapillary pachychoroid syndrome, and pachychoroid pigment epitheliopathy, anatomical improvement was observed after therapy with prednisolone eye drops. Visual acuity did not change significantly.

## Abstract 2

Forty-four eyes with chronic central serous chorioretinopathy, eight eyes with peripapillary pachychoroid syndrome, and two eyes with pachychoroid pigment epitheliopathy were treated with prednisolone eye drops 3 times a day for 6 weeks. Significant anatomical improvement was observed in the eyes with chronic central serous chorioretinopathy and peripapillary pachychoroid syndrome.

The pachychoroid disease spectrum is a compilation of diseases with specific choroidal characteristics. This spectrum includes central serous chorioretinopathy (CSC), pachychoroid pigment epitheliopathy (PPE), pachychoroid neovasculopathy (PNV), polypoidal choroidal vasculopathy/aneurysmal type 1 neovascularization (AT1), focal choroidal excavation, and peripapillary pachychoroid syndrome (PPS).^1^

Pachychoroid disease characteristics are choroidal hyperpermeability on indocyanine green angiography (ICGA), with local thickening of the choroid from dilated choroidal vessels in Haller's layer (pachyvessels) and an attenuated overlying choriocapillaris on optical coherence tomography (OCT).^2^ These features are associated with progressive retinal pigment epithelium (RPE) dysfunction. The precise pathophysiological mechanisms, however, remain unknown. The development of subretinal fluid (SRF) or intraretinal fluid (IRF) and choroidal neovascularization are possible sight-threatening complications.^1^

The most common clinical manifestation of pachychoroid disease is CSC, characterized by a central serous detachment of the neurosensory retina secondary to a decompensated RPE. Acute CSC is a self-limiting disease, with reattachment of the neurosensory retina occurring within 3 to 4 months in the majority of cases.^3^ To prevent irreversible vision loss in patients with longstanding serous retinal detachment, referred to as chronic central serous chorioretinopathy (cCSC), many treatment options have been postulated to reduce the SRF. Frequently performed treatments include photodynamic therapy (PDT), subthreshold micropulse laser treatment, and focal laser photocoagulation in selected cases. The available evidence suggests that half-dose (or half-fluence) PDT is the first-choice treatment in cCSC.^4–7^ However, the deficiency of verteporfin (Visudyne; Alcami Carolinas Corporation, Charleston, SC), required for PDT, has forced ophthalmologists worldwide to search for alternative approaches.

Currently, there is no standard treatment for PPS, as randomized controlled trials are not available. Retrospective case series suggest a beneficial effect of PDT; however, the treatment success rate seems to be lower than in cCSC.^8^

One remarkable treatment option for pachychoroid-related diseases was recently reported in a case series of 17 eyes with chronic retinal fluid due to PPS. Topical treatment with prednisolone eye drops resulted in a reduction of retinal fluid in all cases, although improvement in visual acuity (VA) was inconsistent.^9^ Another study described 10 eyes with recalcitrant CSC treated with topical dexamethasone as an adjuvant to mineralocorticoid receptor (MR) antagonists. Although there was no significant change in VA, 50% of eyes achieved complete resolution of SRF and 30% showed a partial response.^10^

While the use of systemic corticosteroids has been associated with the development of SRF or IRF in patients with CSC,^11,12^ the previous small-scale studies suggest a possible therapeutic role for topical corticosteroids in cCSC and PPS. Patients in this study were treated with prednisolone eye drops, as half-dose PDT could not be performed due to the shortage of verteporfin. We aimed to evaluate the anatomical and functional effect of treating pachychoroid-related diseases with topical prednisolone drops.

## Methods

### Study Design

In this retrospective study, data were collected from the medical records of patients with retinal disease confined to the pachychoroid disease spectrum, treated at Radboud University Medical Centre (Nijmegen, The Netherlands) between September 2020 and February 2023.

### Diagnosis

Diagnosis of pachychoroid-related diseases was based on multimodal imaging, including spectral-domain OCT, OCT angiography (OCT-A), fluorescein angiography, and indocyanine green angiography (ICGA), using the Spectralis HRA + OCT device (SPECTRALIS, Heidelberg Engineering, Heidelberg, Germany).

Pachychoroid was defined as a focal or diffuse increase in choroidal thickness, with pachyvessels in the Haller layer and attenuation of the choriocapillaris.^1^ Diagnosis of PPE was defined as the presence of pachychoroid, with orange-reddish fundus appearance, reduced fundus tessellation, RPE abnormalities with one or more pigment epithelial detachments, and corresponding changes on fundus autofluorescence, but with no history or characteristics indicative of SRF.^13^ cCSC was defined as the presence of SRF with RPE window defects and at least one area of hyperfluorescent leakage on fluorescein angiography, with corresponding hypercyanescent areas on ICGA.^14^ PPS was defined as the presence of peripapillary SRF and/or IRF and RPE alterations. In addition, the choroidal thickness nasally to the fovea had to exceed the temporal choroidal thickness, and pachyvessels had to be present in the nasal choroid on spectral-domain OCT or ICGA.^8,9^

### Patient Selection

Patients with cCSC and PPS who met the following criteria were included: 1) presence of SRF and/or IRF, 2) disease-related symptoms or presence of fluid on spectral-domain optical coherence tomography for at least 3 months or with a recurrence after one or more previous episodes of fluid on SD-OCT, 3) prescription of prednisolone eye drops, and 4) age of 18 years or older. Inclusion criteria for patients with PPE were 1) presence of a vision-threatening pigment epithelium detachment for at least 3 months, 2) prescription of prednisolone eye drops, and 3) age of 18 years or older. Exclusion criteria were the presence of other retinal diseases that can cause SRF or IRF formation; neovascularization visible on OCT angiography (OCT-A); concomitant treatment with anti–vascular endothelial growth factor; treatment within 6 months before the prescription of prednisolone eye drops with one of the following: photodynamic therapy, subthreshold micropulse laser, and focal laser photocoagulation; and prescription of prednisolone eye drops for other purposes (i.e., after surgery, uveitis).

### Treatment

Owing to the deficiency of verteporfin, treatment with PDT could not be performed upon diagnosis. After a thorough explanation of the disease entity and discussion of alternative treatment options and side effects of topical steroids, all included patients consented to off-label treatment with prednisolone acetate 1% eye drops 3 times a day until the follow-up visit, scheduled 6 weeks after treatment initiation. Patients were asked to have their intraocular pressure (IOP) checked by the local optician 10 to 14 days after treatment initiation.

### Data Collection

Clinical data concerning age, sex, extraocular corticosteroid usage, VA, and IOP were obtained from the patients' medical records for both baseline (the moment of treatment initiation) and follow-up visits.

Retinal central subfield thickness was determined using the Heidelberg Eye Explorer software's thickness map with the superimposed Early Treatment of Diabetic Retinopathy Study (ETDRS) grid. Retinal thickness was defined as the distance between the hyperreflective line of the Bruch membrane and the internal limiting membrane. After careful verification of the segmentation, the retinal thickness mentioned within the central 1-mm-diameter circle of the ETDRS grid was recorded as retinal central subfield thickness. Macular volume was measured using the Heidelberg software calculating the retinal volume within the 6-mm-diameter circle of the ETDRS grid. The outer nasal retinal thickness was also obtained using the Heidelberg software measuring the retinal thickness in the outer nasal part of the ETDRS grid. Eyes were classified based on the change of macular volume: decrease in macular volume (reduction of ≥10 mm^3^); stable macular volume (reduction or increase of <10 mm^3^); and increased macular volume (increase of ≥10 mm^3^).

Subfoveal choroidal thickness (SFCT) was measured manually with the caliper function in the Heidelberg Eye Explorer software and was defined as the distance from the hyperreflective line of the Bruch membrane to the chorioscleral border. All measurements were done by two independent investigators (I.M.T. and F.M.v.d.T.) so that every OCT image was evaluated twice. In case of discrepancy between the measurements of the two investigators, images were reevaluated until consensus was obtained.

For evaluation of VA, eyes were classified as follows: increase in VA (improvement of ≥5 letters), stable VA (increase or decrease of <5 letters), and decrease in VA (deterioration of ≥5 letters).

### Statistical Analysis

Statistical analysis was performed using SPSS 27.0 (IBM, SPSS Inc, Chicago, IL). Snellen VAs were converted to ETDRS letter scores as described previously.^15^ Paired *t*-test and Wilcoxon signed rank test were used to analyze continuous variables in case of normal and nonnormal distribution, respectively. McNemar chi-square test was used to analyze categorical variables. The results with a *P* value <0.05 were considered statistically significant (*P* < 0.025 after Bonferroni correction if tested in both cCSC and PPS eyes).

## Results

This study included 54 eyes of 48 patients, with a mean age of 55.0 ± 10.9 years old. Of these, 44 eyes had cCSC, eight eyes had PPS, and two eyes had PPE (Table 1). The mean time from the start of prednisolone eye drops to the follow-up visit was 41.2 ± 14.5 days.

**Table 1.** Patient Characteristics at Baseline

Baseline Characteristics	Total Group (*n* = 48 Patients)
Age, years	55.0 ± 10.9
Sex	
M	41 (85%)
F	7 (15%)
Systemic steroid use at baseline	7 (15%)

	Total Group (*n* = 54 Eyes)
Pachychoroid spectrum disease	
cCSC	44 (81%)
PPS	8 (15%)
PPE	2 (4%)
Mean visual acuity*, ETDRS (Snellen)	68.8 ± 20.3 (20/40)

Data are presented as mean  ±  SD or as absolute numbers and percentages.

* Data available for 53 eyes.

### Prednisolone Eye Drops in Chronic Central Serous Chorioretinopathy

**Fig. 1.**
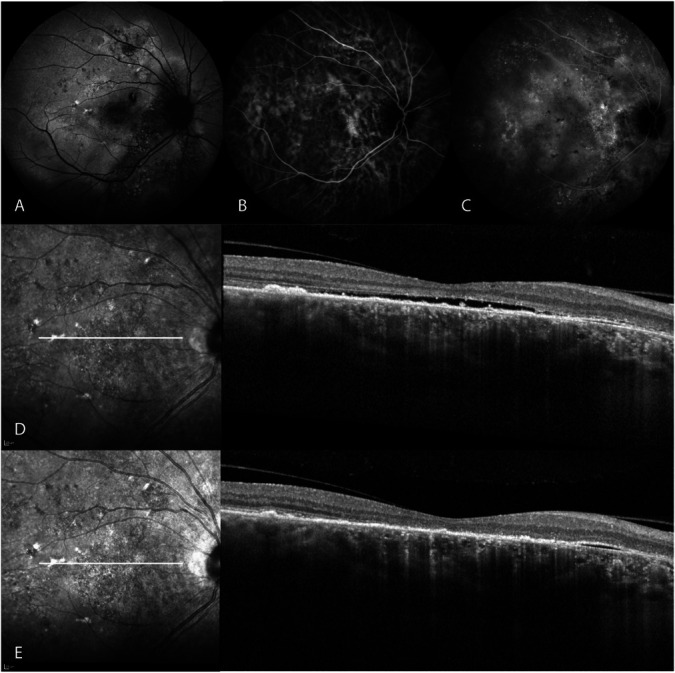
Multimodal imaging of a 67-year-old man with cCSC at baseline (**A**–**D**) and after treatment with prednisolone eye drops for 6 weeks (**E**). At baseline, hypo- and hyperautofluorescence are seen on fundus autofluorescence (**A**). On indocyanine green angiography (**B** and **C**), diffuse hyperpermeability is present. At baseline, subfoveal subretinal fluid is present on OCT (**D**), after treatment with prednisolone eye drops for 6 weeks, subfoveal SRF has resolved (**E**).

In patients with cCSC, the macular volume decreased in 30 eyes (68%), remained stable in 10 eyes (23%), and increased in four eyes (9%). Complete resolution of fluid was seen in four of 44 eyes (9%, *P* = 0.125) and foveal resolution of fluid was seen in nine of 41 eyes (22%, *P* = 0.004) (Figure 1). The macular volume decreased significantly from 8.9 ± 1.1 mm^3^ to 8.5 ± 1.0 mm^3^ (*P* < 0.001), and the retinal central subfield thickness decreased from 330.2 ± 94.8 *µ*m to 294.8 ± 98.4 *µ*m (*P* < 0.001) (Table 2). No significant changes were observed in the subfoveal choroidal thickness. The VA did not change significantly. An improvement of ≥5 letters was seen in eight eyes (19%), 29 eyes remained stable (67%), and six eyes showed a decrease of ≥5 letters (14%). The IOP increased significantly from 14.8 ± 2.8 mmHg to 18.8 ± 5.7 mmHg (*P* < 0.001).

**Table 2.** Clinical and Multimodal Imaging Characteristics at Baseline and After Treatment With Prednisolone Eye Drops

Outcomes	cCSC			PPS		
	Baseline Visit	Follow-up	*P*	Baseline Visit	Follow-up	*P*
Retina						
Macular volume, mm^3^	8.9 ± 1.1 (n = 42)	8.5 ± 1.0 (n = 42)	< 0.001*	8.6 ± 1.2 (n = 8)	8.2 ± 0.7 (n = 8)	0.068†
Retinal central subfield thickness, μm	330.2 ± 94.8 (n = 43)	294.8 ± 98.4 (n = 43)	< 0.001*	269.5 ± 92.6 (n = 8)	243.8 ± 49.4 (n = 8)	0.311†
SRF present	44/44 (100%)	37/44 (84%)	0.016‡	2/8 (25%)	2/8 (25%)	>0.99‡
IRF present	11/44 (25%)	8/44 (18%)	0.250‡	8/8 (100%)	7/8 (88%)	>0.99‡
Foveal SRF and/or IRF present	41/44 (93%)	32/44 (73%)	0.004‡	3/8 (38%)	2/8 (25%)	>0.99‡
SRF and/or IRF present	44/44 (100%)	40/44 (91%)	0.125‡	8/8 (100%)	7/8 (88%)	>0.99‡
Choroid						
SFCT, μm	451.8 ± 115.7 (n = 41)	438.9 ± 130.1 (n = 38)	0.328†	353.7 ± 99.9 (n = 3)	452.5 ± 119.4 (n = 4)	0.283†
Visual acuity, ETDRS (snellen)	68.8 ± 20.8 (20/40) (n = 43)	68.6 ± 20.3 (20/40) (n = 44)	0.800*	66.6 ± 20.4 (20/50) (n = 8)	68.6 ± 23.7 (20/40) (n = 8)	0.519†
IOP, mmHg	14.8 ± 2.8 (n = 40)	18.8 ± 5.7 (n = 36)	<0.001*	15.4 ± 3.0 (n = 7)	22.5 ± 7.0 (n = 8)	0.043†
IOP ≥25 mmHg	0/40 (0%)	6/36 (17%)	0.063‡	0/7 (0%)	2/8 (25%)	0.500‡

Outcome measures are presented in mean ± SD or as absolute numbers and percentages. Percentages were calculated using nonmissing data.

A Bonferroni correction was performed, and values were considered significant at *P* < 0.025 after correcting for repeated testing.

Retinal thickness and macular volume were determined based on the distance between the internal limiting membrane and the hyperreflective line of Bruch membrane.

* Wilcoxon signed rank test.

† Paired *t*-test.

‡ McNemar chi-square test.

### Prednisolone Eye Drops in Peripapillary Pachychoroid Syndrome

**Fig. 2.**
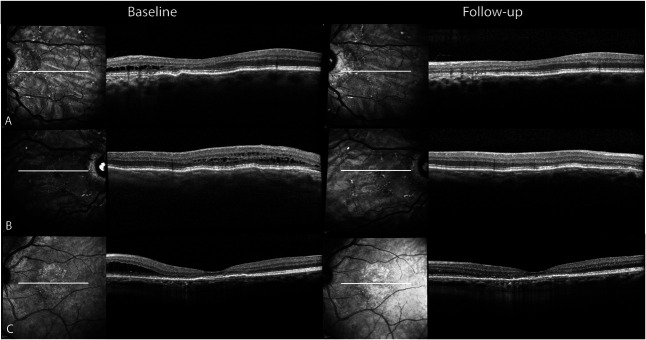
Optical coherence tomography in three cases with PPS before and after treatment with prednisolone eye drops (**A**–**C**). In all presented cases, a partial or a complete resolution of intraretinal fluid was observed at a follow-up of 5 to 8 weeks. In case C, only the horizontal scan through the fovea is depicted. The most prominent thickening of the nasal choroid was visible more superiorly (not shown), in accordance with the diagnosis of PPS.

Total macular volume and retinal central subfield thickness in PPS did not change significantly between the baseline visit and the follow-up visit (Table 2). The outer nasal retinal thickness decreased from 328.0 ± 55.8 *µ*m to 295.8 ± 29.5 *µ*m (*P* = 0.025). Macular volume decreased in six eyes, remained stable in one eye, and increased in one eye. Complete resolution of fluid was seen in one of eight eyes (Figure 2, Table 2). Visual acuity and subfoveal choroidal thickness did not change significantly. Visual acuity increased with ≥5 letters in two eyes, remained stable in four eyes, and decreased by ≥5 letters in two eyes. A nonsignificant rise in IOP was measured.

### Prednisolone Eye Drops in Pachychoroid Pigment Epitheliopathy

**Fig. 3.**
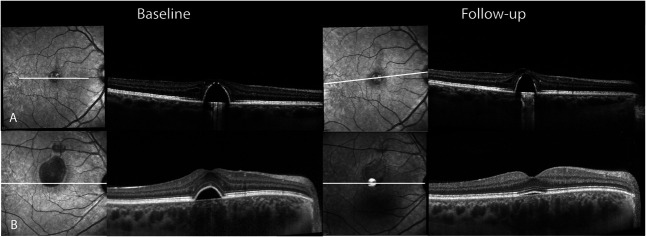
Optical coherence tomography before and after treatment with prednisolone eye drops in patients with PPE (**A** and **B**). In one patient with a longstanding pigment epithelial detachment with secondary pigment epithelium atrophy and accompanying intraretinal fluid (**A**), no decrease in height and width of the pigment epithelial detachment was seen at follow-up. In the other patient (**B**), a decrease in the height and width of the pigment epithelial detachment was seen.

In one of the two patients with PPE, a decrease in the height of the pigment epithelial detachment was visible after treatment with prednisolone eye drops. In the other patient, there was no difference in the height of the pigment epithelial detachment (Figure 3). Visual acuity remained stable in both eyes with PPE.

## Discussion

This study represents the largest retrospective cohort described hitherto investigating the effect of steroid eye drops in pachychoroid-related disease. The majority of included eyes suffered from cCSC. Significant anatomical response was seen in cCSC and PPS cases.

Considering the patients with cCSC, a significant reduction in total macular volume and retinal central subfield thickness after use of steroid eye drops was found, due to a decrease in sub- or intraretinal fluid. Complete resolution of fluid was seen in four of 44 eyes (9%), and resolution of foveal fluid occurred in nine of 41 eyes (22%) at follow-up. In comparison, complete resolution of SRF was observed in two of 54 (4%) eyes in the placebo group after 4 weeks in the VICI trial, a double-blind placebo-controlled trial in which oral eplerenone treatment was compared with placebo in patients with cCSC.^16^ Another recent report on 10 patients with recalcitrant CSC treated with dexamethasone eye drops combined with an oral MR antagonist showed complete resolution of SRF in one of 10 eyes after one month and in five of 10 eyes after three months.^10^ The higher percentage of patients with complete resolution after three months might be explained by the longer follow-up time. Prolonged treatment with steroid eye drops in our patients showing already good, but incomplete, response at 1 month might result in complete resolution of fluid. Anyhow, it remains a surprising observation that topical steroids in our patients with cCSC resulted in decreased macular volume in 68% in contrast to increased macular volume in only 9% of eyes, considering the known association of extraocular corticosteroid use with the development of cCSC.

No significant change in VA was observed in our patients with cCSC. This was to be expected considering the longstanding presence of subretinal fluid and widespread outer retinal changes already present at the baseline visit. Owing to the shortage of verteporfin, treatment with PDT was delayed in patients with cCSC. A previous report showed no difference in BCVA between baseline and last follow-up visit after a delay in PDT, however, a deterioration of ≥5 letters was seen in 30.5% of the eyes.^17^ Therefore, in the absence of PDT, prednisolone eye drops treatment potentially prevents additional visual loss in a portion of the patients with cCSC by reducing the duration of subretinal fluid.

Considering the eight cases with PPS treated with steroid eye drops, no significant change in central retinal thickness, total macular volume, and VA was observed. However, the fluid in PPS is centered around the optic disk, and, therefore, total retinal macular volume and central retinal thickness are suboptimal measures to evaluate the treatment effect in this group. Pothof et al analyzed 17 eyes with PPS treated with prednisolone eye drops. They reported a reduction of peripapillary intraretinal fluid in all 17 eyes after 4 weeks of treatment, of which seven eyes (41%) showed complete resolution of fluid.^9^ When analyzing the outer nasal retinal thickness in the ETDRS grid in this study, a significant reduction was found (*P* = 0.025).

Since there were only two patients with PPE included, no valid conclusions could be drawn from their results. Nevertheless, one patient showed clear anatomical improvement after treatment with prednisolone eye drops.

In the patients with cCSC, IOP significantly increased after treatment, and 17% of eyes had an IOP > 25 mmHg at follow-up, which does not exceed the reported incidence of steroid-induced ocular hypertension in the general population.^18^ In the patients with PPS, a nonsignificant rise in IOP was measured. The increase in IOP could be one explanation for the observed reduction of SRF or IRF. It is however unlikely that this fluid reduction is caused by the mechanical effect of an increased IOP pushing the retina toward the choroid since several patients with reduction in intra- or subretinal fluid did not have an IOP elevation. Nevertheless, the risk of steroid-induced ocular hypertension is an evident disadvantage of prednisolone eye drops and should be discussed with the patient before initiating treatment.

The pathophysiology of pachychoroid disease remains elusive, but several mechanisms have been suggested previously. According to the venous overload theory, fluid leakage from the choroid is caused by increased intraluminal pressure in the choriocapillaris, due to downstream venous overload from an increased resistance at the level of the ampulla.^19^ Alternatively, the presence of pachyvessels might be caused by increased arterial inflow, due to preexisting or acquired choroidal arteriovenous anastomoses.^20^ However, these theories do not provide answers as to the role of corticosteroids in the disease process, despite the observation that the use of extraocular corticosteroid medication is one of the most consistent risk factors associated with CSC flare-ups.^11,12^ Even more intriguing is the finding that ocular steroid use, as opposed to extraocular, may reduce the sub- or intraretinal fluid, as seen in this study. There are several possible mechanisms that could explain this result. One hypothesis is that the anti-inflammatory effect of topical steroids causes a reduction in retinal fluid. However, this seems less likely because one would also expect an anti-inflammatory effect from systemic corticosteroids, and the latter are associated with the development of retinal fluid. Furthermore, MR overactivation could play a role in CSC, causing vasodilation and leakage in the choroid.^21,22^ However, in the VICI trial, oral therapy with an MR antagonist was not superior to placebo neither functionally nor anatomically.^16^ One study found a decrease in intraocular cortisol levels in patients with CSC compared with unaffected controls, and an imbalance of the MR/glucocorticoid receptor pathway was suggested.^23^ In a review article by Bousquet et al, combination therapy of an MR antagonist with topical corticosteroids was suggested, to simultaneously block the MR and stimulate the glucocorticoid receptor, thereby restoring the MR/glucocorticoid receptor equilibrium.^24^ In our study, topical corticosteroids alone seemed to be effective in reducing subretinal fluid in cCSC.

We recognize the limitations of this study, especially its retrospective design, the lack of a control group, and the short follow-up time. Randomized controlled trials investigating the efficacy and safety of this treatment option are required, especially considering the natural course of cCSC involving spontaneous fluid reduction.

In conclusion, this study suggests that topical prednisolone eye drops may serve as an alternative treatment for a subset of nonneovascular pachychoroid-related diseases. Although the mode of action is still elusive, there is currently an ongoing shortage of verteporfin and a subsequent lack of what is considered the best treatment for patients with cCSC, namely PDT. Therefore, prednisolone eye drops could be used to bridge the gap until verteporfin becomes widely available again and prevent unnecessary vision loss in a subset of patients. Furthermore, prednisolone eye drops may serve as a low-cost and easily accessible treatment alternative in countries where PDT is not available at all or where PDT is not being reimbursed. Although additional studies are warranted, we conclude that topical steroidal eye drops may be considered as a treatment option for cCSC and PPS.
